# Analysis of Risk Factors Related to Carotid Artery in Patients with Acute Ischemic Stroke

**DOI:** 10.2174/0115734056320499260424094133

**Published:** 2026-04-28

**Authors:** Dandan Xiao, Hongjun Nie, Yaocheng Xiao, Liyun Chen, Yanfen Zhang

**Affiliations:** 1 Department of Ultrasound, The Affiliated Changsha Central Hospital, Hengyang Medical School, University of South China, Changsha, 41000, China

**Keywords:** Carotid plaque, Contrast-enhanced ultrasound, New blood vessels in plaque, Acute ischemic stroke, Intraplaque neovascularization, Transient ischemic attack

## Abstract

**Objective:**

Studying independent risk factors for carotid artery-related Acute Ischemic Stroke (AIS) in affected patients can help guide the clinical prevention and prognosis of AIS.

**Methods:**

In this retrospective study, 81 patients who were admitted to our center for routine carotid ultrasound and contrast-enhanced ultrasound examinations were enrolled. The patients were assigned to the study and control groups based on whether they had AIS symptoms. Multivariate logistic regression was used to analyze the correlation between risk factors and carotid artery-related AIS.

**Results:**

Significant differences in Intraplaque Neovascularization (IPN) grade, vascular stenosis, different age stages, plaque length and diameter, and hypertension were observed between the two groups (*P* < 0.05). Two sonographers were satisfactorily consistent in IPN grading diagnosis (Kappa = 0.763). According to the multivariate logistic regression analysis, the IPN grade and hypertension were independent risk factors for carotid artery-associated AIS (*P* < 0.05). Receiver Operating Characteristic (ROC) analysis showed that IPN grading demonstrated better discriminative performance for AIS than lumen stenosis, with an Area Under the Curve (AUC) of 0.74 versus 0.65.

**Discussion:**

In the study group, the carotid plaques of AIS patients were mostly of IPN grade III-IV. The number of patients with IPN > II was significantly higher in the study group than in the control group (33.3% (27/81) *vs*. 7.4% (6/81); *P* < 0.05). The accuracy, sensitivity, specificity, and positive and negative predictive values of carotid canal cavity stenosis were approximately 65.43%, 64.29%, 66.67%, 67.50%, and 63.41%, respectively.

For patients with IPN > II, the values for the aforementioned parameters were 76.54%, 81.81%, 72.92%, 85.36%, and 67.50%, respectively. Statistically significant differences in sensitivity and negative predictive value were observed between the two groups (*P* < 0.05).

**Conclusion:**

IPN grading demonstrates a stronger association and higher discriminative ability for AIS than for carotid stenosis. It may provide valuable information for early clinical identification, risk stratification, and prevention of carotid artery-related AIS.

## 1 INTRODUCTION

Acute Ischemic Stroke (AIS) is the most common type of stroke. The rate of AIS-related mortality has recently been increasing rapidly in China [1]. This increase is related to the increasing prevalence of cardiovascular diseases (such as hyperlipidemia, diabetes, and hypertension). Stroke is the second leading cause of death worldwide and the main cause of long-term acquired disability [2]. Cerebral computed tomography examination in ischemic stroke patients may reveal no positive manifestations within 24 h or may exhibit atypical low-density areas. T1 and T2 may also exhibit no signal changes during the hyperacute period of brain magnetic resonance imaging for 6 hours. If the exact imaging evidence is not obtained, ischemic stroke is diagnosed on the basis of clinical signs that last for more than 24 h. Early AIS identification is very crucial for the early treatment and prevention of this type of stroke.

The carotid artery is the main source of blood supply to the brain, and the vulnerable carotid atherosclerosis plaque is among the main causes of AIS [3]. The ultrasonic characteristics of carotid plaques can be determined through routine ultrasound examinations, and plaque vulnerability can be judged to some extent. Contrast-Enhanced Ultrasound (CEUS) examination offers greater advantages in assessing plaque vulnerability. Intraplaque Neovascularization (IPN) is closely related to vulnerable plaques. CEUS can reflect the distribution of plaque neovascularization through microbubbles [4, 5]. Evaluating carotid plaques using CEUS is of great research value in the present evidence. Studies worldwide have evaluated the relationship between ischemic stroke and carotid IPN [6, 7]. However, only a few studies have evaluated whether carotid IPN plays an independent role in carotid artery-related AIS regardless of other factors (such as age, sex, hyperlipidemia, diabetes, hypertension, and ultrasonic characteristics of carotid plaques) [8].

We performed multivariate logistic regression analysis to analyze related clinical data, laboratory examination indicators, imaging data, carotid IPN CEUS grading, other influencing factors, *etc*., and discussed the influencing factors and their correlation with patients with carotid-related AIS.

## 2 MATERIALS AND METHODS

### 2.1 General Information

This single-center retrospective study screened 96 patients admitted to Changsha Central Hospital, Hunan Province, between November 2021 and March 2023 who were found to have carotid plaques on routine carotid ultrasound. Fifteen patients were excluded, including 7 who did not undergo Contrast-Enhanced Ultrasound (CEUS), 5 with incomplete data, and 3 with a history of prior stroke or Transient Ischemic Attack (TIA). The remaining 81 patients were consecutively enrolled and underwent both carotid ultrasound and CEUS examination. The study population included 66 men and 15 women aged 46-87 years (average age: 65.33 ± 8.15 years). All patients also underwent cranial imaging examinations. Patients were eligible if carotid plaques were detected on routine ultrasound with a thickness ≥ 1.5 mm, and CEUS was performed to further evaluate plaque characteristics. Baseline clinical information was extracted from medical records, including smoking status, alcohol consumption, body mass index, family history of cardiovascular disease, and laboratory indicators. In this cohort, no patients had a history of smoking, obesity, or a family history of cardiovascular disease, while some patients reported alcohol consumption. Lipoprotein subfractions and homocysteine levels were within normal ranges and therefore were not included in the regression analysis.

**Fig. (1):**
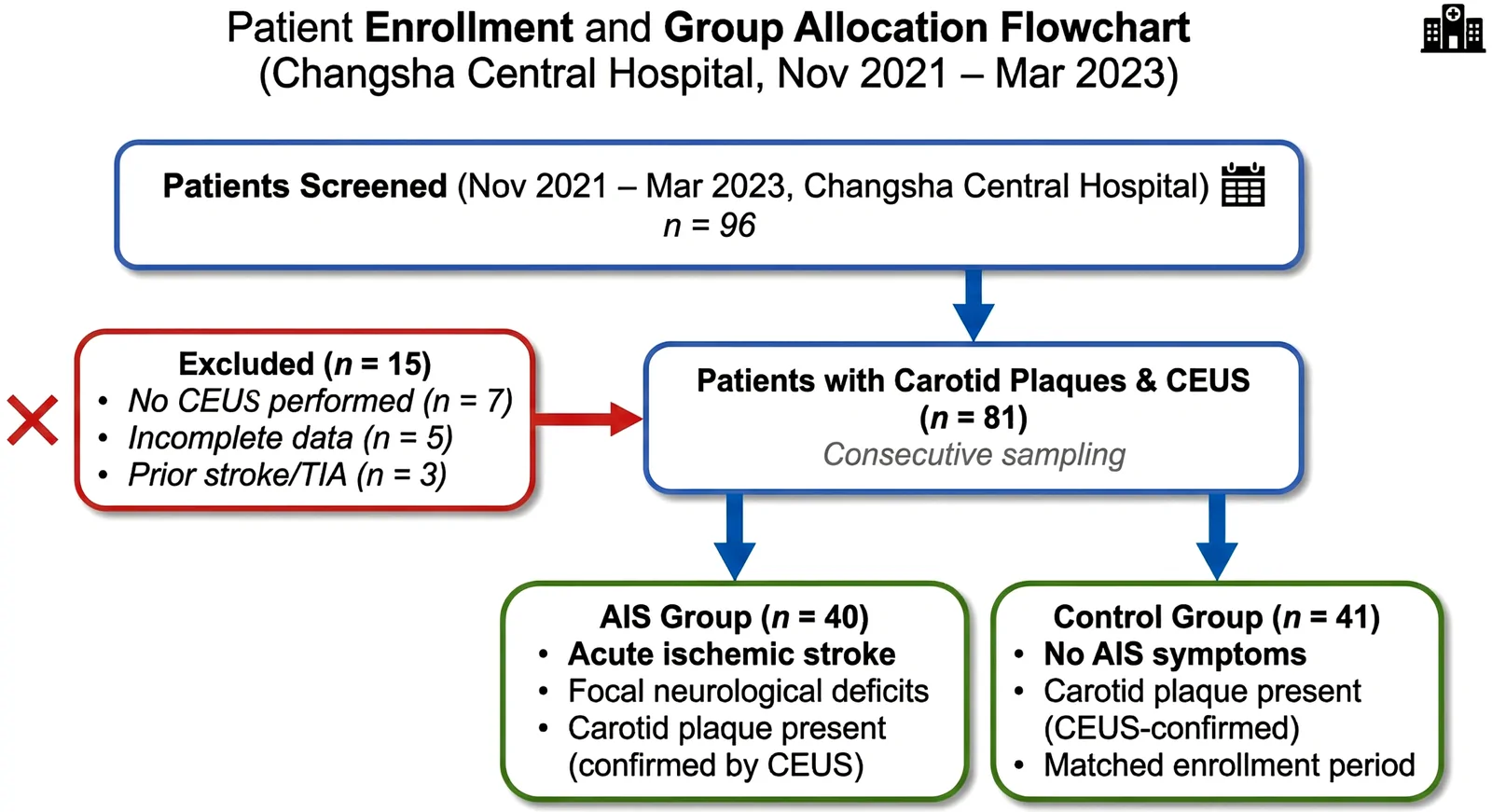
STROBE flow diagram showing participant selection, exclusion criteria, and group allocation.

Patients who met the criteria of *China Guidelines for the Diagnosis and Treatment of Acute Ischemic Stroke* (*2018 Edition*) [9] and presented with focal neurological deficits attributable to ischemic stroke on the responsible side (*e.g*., limb weakness, numbness, limited mobility, or facial asymmetry) were assigned to the AIS group (n = 40). Patients with carotid plaques but without AIS during the same period served as controls (n = 41). Cerebrovascular diseases such as previous stroke, transient ischemic attack, and acute cerebral hemorrhage were excluded to confirm carotid artery-related AIS. The participant screening and group allocation are illustrated in the STROBE flow diagram (Fig. **1**).

The patient exclusion criteria were as follows: patients with definite intracranial vascular malformation, those who could not cooperate due to severe mental illness; those with severe heart, liver, and kidney diseases; those with severe bleeding tendency; those for whom complete and relevant clinical data, laboratory examination indicators, and imaging data were not available; those with a history of stroke, such as old cerebral infarction or sequelae of previous cerebral infarction; and those with transient ischemic attack and acute cerebral hemorrhage were not included in the study group. The ethics committee of our hospital approved the study, and all patients provided informed consent.

Relevant clinical data, laboratory examination indices (including gender, age, diabetes history, hypertension, and blood lipid), skull imaging examination data, routine carotid ultrasound (location, size, echo, and degree of vascular stenosis) data, and CEUS data related to carotid plaque neovascularization were collected to analyze independent risk factors for patients with carotid artery-associated AIS. The data were all obtained during hospital admission.

### 2.2 Instrument

This study used the Philips EPIQ5 ultrasonic diagnostic instrument equipped with an ultrasound contrast function and a L9-4 linear array probe set to a frequency of 4-9 MHz. The SonoVue contrast agent (8 mg/mL) and physiological saline (0.9% NaCl solution) were injected through the instrument.

### 2.3 Methods

In the routine carotid ultrasound examination, the patient was asked to lie flat with his head slightly backward, exposing his neck. During the examination, the patient’s head was slightly leaned to one side. The scanner scanned the common carotid artery horizontally and vertically, thereby focusing on carotid plaques and their surrounding vascular segments; saving gray-scale ultrasound, color Doppler, and pulse Doppler images; measuring the maximum plaque size; and recording the echo characteristics of plaque. During the CEUS examination, to prepare the contrast agent, 5 mL of 0.9% sodium chloride solution was injected into the SonoVue freeze-dried powder bottle and shaken evenly. The patients were instructed to breathe calmly and avoid violent actions such as coughing and swallowing, and the contrast mode was entered. The mechanical index of the probe was adjusted to 0.1, and the carotid plaque was selected as the region of interest for observation. Then, 2 mL of the contrast agent was quickly injected through the right elbow vein. A dynamic video was recorded and stored for further analysis.

The IPN grading of CEUS was as follows [10]: Grade I: no microbubbles are noted in the plaque; Grade II: a small number of microbubbles are observed in the plaque, which are distributed on the plaque’s outer membrane side; Grade III: linear microbubbles are displayed in the plaque, which are on the shoulder or in the plaque and do not extend to the center or top of the plaque; and Grade IV: numerous microbubbles are present in the plaque. Based on this grading system, two sonographers with rich experience in contrast-enhanced imaging judged the plaque grade, and the consistency of the two diagnoses was verified through the Kappa test.

### 2.4 Statistical Analysis

The sample size was based on all consecutive eligible patients. For logistic regression, ≥ 10 events per variable are recommended; 40 AIS events were allowed for stable inclusion of predictors. With n = 81, the study has > 80% power to detect an OR ≥ 2 at α = 0.05.

SPSS 26.0 was used for statistical analysis, and normally distributed measurement data were represented by ± s. The two independent-samples T test was performed for comparing the groups, and non-normally distributed measurement data were represented by M (QR). The Kruskal-Wallis H test was used for comparing the groups. The counted data were expressed by n (%), and the between-group comparisons were made using the χ^2^ test. AIS was considered the dependent variable, and age, sex, hyperlipidemia, diabetes, ultrasonic evaluation of carotid plaques, and other influencing factors were considered as independent variables. The independent risk factors for carotid artery-related AIS and the correlation between each risk factor and AIS were evaluated through the multivariate logistic regression analysis. The difference of *P* < 0.05 was considered statistically significant. Receiver Operating Characteristic (ROC) curve analysis was performed to evaluate the discriminative performance of IPN grading and lumen stenosis for identifying AIS. The Area Under the Curve (AUC), sensitivity, and specificity were calculated.

## 3 RESULTS

### 3.1 Comparison of Baseline Data of the Two Groups

In total, 81 patients were enrolled in the study, including 40 in the experimental group (men: 35 and women: 5; age: 46-87 years; average age: 66.80 ± 8.51 years). The control group included 41 patients (31 men and 10 women; age 51-79 years; average age: 63.90 ± 7.62 years). No significant difference in age and sex was observed between the two groups (*P* > 0.05). The study followed the Sex and Gender Equity in Research guidelines (SAGER) to ensure appropriate inclusion and reporting of sex-related data.

### 3.2 Comparative Analysis of Clinical Data, Laboratory Examination Indices, and Imaging Examination Data Between the Two Groups

No significant differences in sex, dyslipidemia, diabetes, plaque location, plaque thickness, and echo were observed between the study and control groups (*P* > 0.05). However, significant differences were noted in IPN grade, vascular stenosis, different age stages, plaque length and diameter, and hypertension between the two groups (*P* < 0.05; Tables **1** and **2**).

### 3.3 Contrast Analysis of IPN Grading of Carotid Artery CEUS Between the Two Groups

The χ^2^ test was conducted to compare the study and control groups, and a statistical difference was noted between the two groups (*P* < 0.05). The study group mostly included patients with IPN grade III-IV. IPN grade > II accounted for 33.3% (27/81) of the patients, which was considerably higher than the percentage of patients with IPN grade > II in the control group (7.4%, 6/81) (Table **3**).

### 3.4 Carotid Canal Cavity Stenosis and IPN Classification Comparison

The accuracy, sensitivity, specificity, and positive and negative predictive values of carotid canal cavity stenosis were approximately 65.43%, 64.29%, 66.67%, 67.50%, and 63.41%, respectively. For patients with IPN > II ^11^, the diagnostic accuracy, sensitivity, specificity, and negative predictive value were better than those of carotid canal stenosis, which were 76.54%, 81.81%, 72.92%, and 85.36%, respectively. The sensitivity (81.81% and 64.29%, respectively, *P* = 0.004) and negative predictive value (85.36% and 63.41%, respectively, *P* < 0.001) of the two groups were statistically different (*P* < 0.05) (Tables **4**-**6**).

### 3.5 Analysis of the Influence of Different Factors on AIS Based on the Logistic Regression Model

AIS occurrence was considered a dependent variable, and IPN grade, degree of vascular stenosis, age, plaque length, and hypertension were considered as independent variables. Logistic regression analysis revealed that IPN grade and hypertension were independent risk factors for AIS, with the difference being statistically significant (*P* < 0.05). Age showed a borderline association with AIS (P = 0.053) and was therefore not considered an independent risk factor. The higher the IPN grade, the greater the AIS risk, even after adjusting for vascular stenosis, age, plaque length, and hypertension (OR: 3.135, 95% CI: 2.212-4.503, *P* < 0.001, Table **7**).

### 3.6 Diagnostic Performance of IPN and Stenosis for AIS

**Fig. (2):**
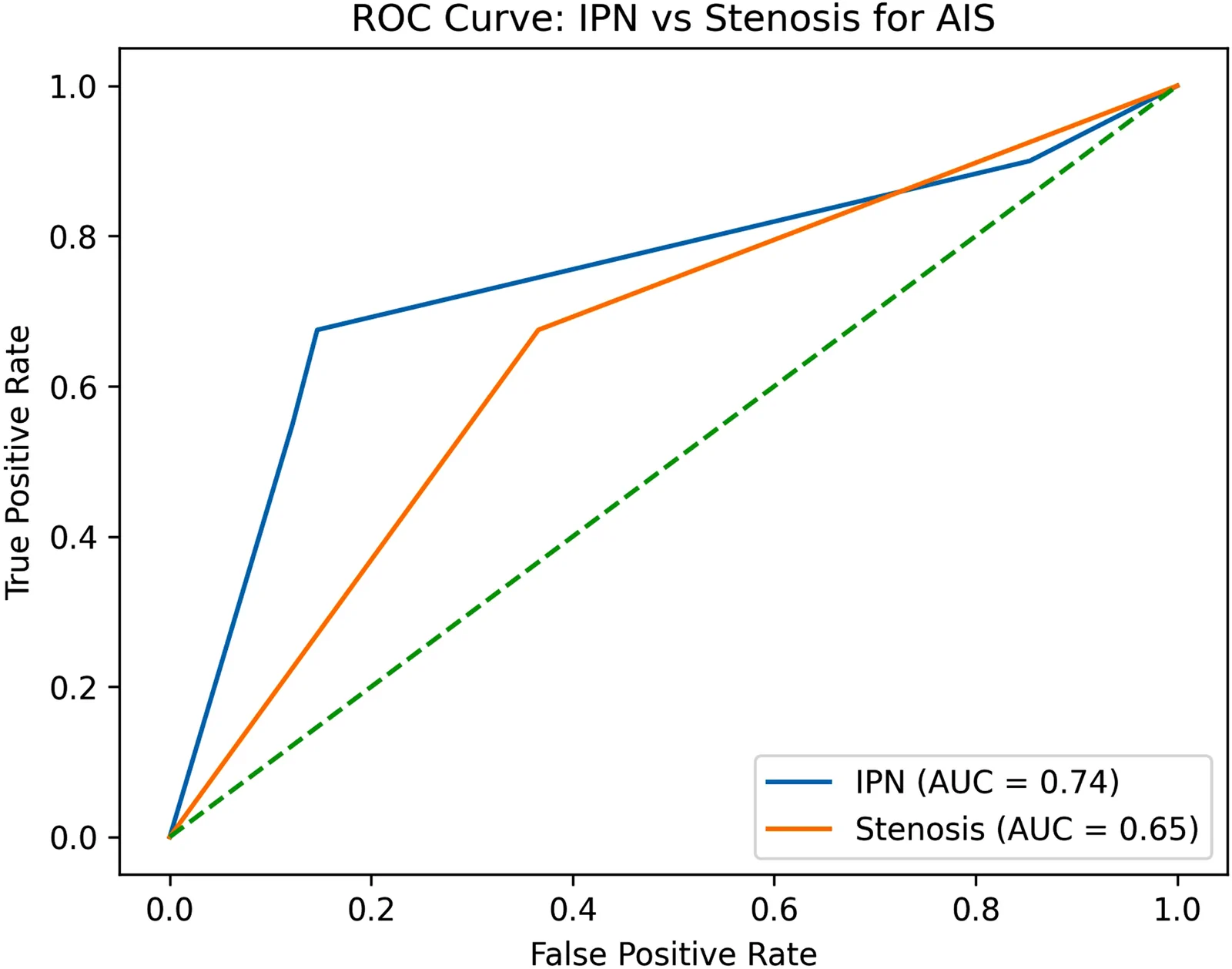
Receiver Operating Characteristic (ROC) curves comparing the diagnostic performance of Intraplaque Neovascularization (IPN) grading and lumen stenosis for identifying Acute Ischemic Stroke (AIS). IPN demonstrated a higher area under the curve (AUC = 0.74) than stenosis (AUC = 0.65), indicating superior discriminative ability.

ROC analysis demonstrated that IPN grading showed better discriminative performance for AIS than lumen stenosis. The AUC for IPN grading was 0.74, compared with 0.65 for stenosis (Fig. **2**). These findings indicate that IPN provides superior diagnostic accuracy for identifying AIS risk.

## 4 DISCUSSION

Ischemic stroke accounts for approximately 70% of all strokes worldwide, and AIS, also referred to as acute cerebral infarction, represents a major global health burden. In patients with carotid atherosclerosis, IPN has been associated with plaque vulnerability and ischemic stroke recurrence [11, 12]. IPN originates from neovessels in the adventitia of the carotid artery and may extend toward the intima [13]. A significant correlation has been observed between microbubble enhancement on CEUS and intraplaque neovascularization and inflammation associated with IPN may contribute to intraplaque hemorrhage and plaque instability [14]. CEUS enables sensitive visualization of plaque neovascularization by detecting microbubble perfusion. Neovascularization may extend from the adventitia to the plaque shoulder and core as plaque progression occurs [15,16]. Microbubble enhancement within carotid plaques on CEUS reflects intraplaque perfusion and has been reported to be associated with cerebral infarction risk, consistent with the findings of the present study.

AIS refers to acute onset stroke, wherein focal lesions are observed on the responsible side, as observed through brain imaging, or clinical signs of related focal neurological dysfunction last for more than 24 hours [9]. Carotid atherosclerosis, which is mainly caused by high carotid stenosis and vulnerable plaque, is a common cause of AIS [17]. Carotid-related AIS is of great public health concern because of its poor clinical prognosis [18]. In the present study, 81 patients were included. The study group patients (n = 40) were mostly from our hospital and had acute onset stroke, which was very representative of carotid artery-related AIS. Among them, 17 patients (42.5%) had negative findings on brain imaging, reflecting the clinical complexity of AIS diagnosis and enhancing the representativeness of carotid artery-related AIS in this cohort.

### 4.1 Positive Factors of AIS

Comparative analysis of clinical, laboratory, and imaging data in patients with carotid artery-related AIS revealed no significant differences between the AIS and control groups in sex, dyslipidemia, diabetes, plaque location, plaque thickness, or echogenicity (*P* > 0.05). However, significant differences were observed in IPN grade, degree of vascular stenosis, age distribution, plaque length and diameter, and hypertension (*P* < 0.05). A higher proportion of patients aged > 65 years was observed in the AIS group (68.9%) compared with the control group (25.5%). Age showed a borderline association with AIS in multivariable analysis (P = 0.053) and therefore should not be interpreted as an independent risk factor. Previous studies have reported that advanced age is associated with poorer functional outcomes and reduced treatment response in patients with AIS [9]. These findings highlight the importance of careful risk assessment and secondary prevention in elderly patients with a potential stroke risk. In the present study, plaque length showed a stronger association with AIS than plaque thickness. From a hemodynamic perspective, the stress point is at the shoulder, and most plaque shoulders are more likely to cause rupture and thrombosis [19]. The longer the stress surface of the plaque is, the more likely it is to rupture or undergo thrombosis. Although plaque thickness also affects plaque stability, stability also depends on factors such as whether the ratio of the thickness to the original plaque diameter causes stenosis of the local lumen.

In routine carotid ultrasound examinations, stenosis is commonly assessed according to the NASCET measurement standard [20], calculated as (internal carotid artery diameter-minimum residual diameter)/internal carotid artery diameter ×100%. Based on the stenosis degree, stenosis can be categorized as mild (< 50%), moderate (50%-69%), or severe. In the present study, IPN > II was more frequently observed in the AIS group than in the control group (P < 0.05). Compared with lumen stenosis, IPN > II showed higher sensitivity and negative predictive value for identifying carotid artery-related AIS. However, intracranial collateral circulation and compensatory cerebral blood flow may maintain perfusion despite carotid stenosis, which may partly explain why AIS does not occur in all patients with significant stenosis [21, 22]. Additionally, in asymptomatic and symptomatic patients with a low degree of carotid artery stenosis, CEUS with microbubbles can more sensitively depict the contour of the hypoechoic plaque [23], thereby supplementing the results of carotid color Doppler and pulse Doppler in many dimensions. This would thus improve the diagnostic value of ultrasound in the carotid plaque. Therefore, the stenosis degree is one-sided in predicting carotid artery-related AIS, which is consistent with the results of previous studies [23-26]. In the present study, IPN > II was significantly more frequent in the AIS group than in the control group (P < 0.05). Compared with lumen stenosis, IPN > II demonstrated higher sensitivity and negative predictive value for identifying carotid artery-related AIS. These findings suggest that IPN reflects plaque vulnerability and may provide additional diagnostic information beyond the degree of stenosis.

Further, multivariate logistic regression analysis revealed that IPN grade and hypertension were independently associated with carotid artery-related AIS. According to the results of the special epidemiological survey of stroke in China, hypertension is the primary risk factor for stroke survivors [12]. Every 10 mmHg increase in systolic blood pressure increases the stroke risk by 53% [26]. This may be because a sustained rise in blood pressure can easily damage the intima of blood vessels. Second, as the pressure on the wall of blood vessels increases, external forces are more likely to distort plaques, thereby rupturing vulnerable plaques and inducing AIS. Active management and monitoring of hypertensive patients are beneficial for the prevention and treatment of AIS.

In addition, ROC analysis demonstrated that intraplaque neovascularization assessed by CEUS showed better discriminative ability for AIS than lumen stenosis alone. This finding supports the role of IPN as an imaging marker of plaque vulnerability and suggests that incorporating IPN assessment may improve risk stratification in patients with carotid atherosclerosis.

## 5 LIMITATIONS

This study has several limitations. First, it was a single-center retrospective study involving a relatively small sample size, which may introduce selection bias and limit the generalizability of the findings. In addition, the absence of external validation and the observational design may allow for residual confounding and the possibility of reverse causation. Second, laboratory data were obtained from a single routine examination after admission and therefore may not fully reflect dynamic changes before and after treatment. Third, the grading of Intraplaque Neovascularization (IPN) using CEUS was based on a semi-quantitative and ordinal assessment, which may introduce subjectivity and limit mechanistic interpretation; future studies incorporating quantitative perfusion parameters may provide more precise evaluation. Finally, long-term outcomes were not assessed due to incomplete follow-up. Future multicenter prospective studies with larger sample sizes, comprehensive clinical data collection, and standardized follow-up are warranted to validate and extend these findings.

## CONCLUSION

According to our findings, carotid IPN CEUS grading, which is IPN grading > II, has a better sensitivity and negative predictive value in predicting carotid artery-related AIS than carotid canal stenosis. This may help improve the prediction of AIS risk and facilitate early prevention.

In this retrospective cohort of patients with carotid atherosclerotic plaques, intraplaque neovascularization assessed by contrast-enhanced ultrasound demonstrated a stronger association and better discriminative performance for acute ischemic stroke than lumen stenosis. IPN grades > II showed higher sensitivity and negative predictive value, and ROC analysis confirmed superior diagnostic accuracy. These findings suggest that IPN may serve as an imaging marker of plaque vulnerability and may aid AIS risk stratification. Prospective multicenter studies are warranted to validate these findings.

## Figures and Tables

**Table 1:** Comparative analysis of data between the research and control groups.

**Group**	**No.**	**Age years (No)**						**Male** **(No)**	**Dyslipidemia (No)**	**Hypertension (No)**	**Diabetes (No)**	**Location (No)**	
		**<55**	**55-59**	**60-64**	**65-69**	**70-74**	**>74**					**Right**	**Left**
Control	41	5 (12.2%)	5 (12.2%)	15 (36.6%)	8 (19.5%)	2 (4.9%)	6 (14.65%)	30 (45.5%)	21 (52.5%)	20 (52.5%)	16 (53.3%)	16 (39.0%)	25 (61.0%)
Experimental	40	1 (2.5%)	11 (27.5%)	2 (5.0%)	9 (22.5%)	11 (27.5%)	6 (15.0%)	36 (54.5%)	19 (47.5%)	27 (47.5%)	14 (46.7%)	17 (42.5%)	23 (57.5%)
x^2^/t/H		21.138						3.800	0.112	2.913	0.042	0.101	
*P*-value		0.001						0.051	0.738	0.088	0.838	0.750	

**Table 2:** Comparative analysis of IPN grading of carotid artery contrast-enhanced ultrasound between the two groups.

**Group**	**No.**	**Plaque size(mm) (No, %)**		**Plaque echo (No)**				**IFN grade (No)**				**Degree of vascular lumen stenosis (No)**			
		**length**	**Thick diameter**	**Low echo**	**Equal echo**	**Heterogeneous echo**		**I**	**II**	**III**	**IV**	**0%**	**<50%**	**50-69%**	**70-99%**
Control	41	15.2 (7.2%)	3.7 (1.4%)	7 (18.1%)	26 (60.6%)	8 (21.2%)		6 (15.2%)	29 (72.7%)	1 (3.05%)	5 (12.1%)	26 (60.1%) (60.1)	7 (21.2%)	6 (12.1%)	2 (6.1%)
Experimental	40	17.25 (13.88%)%)	3.85 (1.64%)	9 (21.9%)	20 (59.4%)	11 (18.8%)		4 (6.3%)	9 (28.1%)	5 (15.6%)	22 (50.0%)	13 (34.4%) (34.4)	14 (37.5%)	9 (15.6%)	4 (12.5%)
x^2^/t/H		18.695	3.374	1.494			24.288						7.922		
*P*-value		<0.001	0.066	0.474			<0.001						0.048		

**Table 3:** Comparison of the proportion of different grades of neovascularization in carotid plaque between the two study groups.

**Group**	**I (IFN) No, %**	**II (IFN) No, %**	**III-IV (IFN) No, %**	**Total No, %**
Control	6(7.4%)	29(35.8%)	6(7.4%)	41(50.6%)
Experimental	4(4.9%)	9(11.1%)	27(33.3%)	40(49.4%)
Total	10(12.3%)	38(46.9%)	33(40,7%)	81(1)

**Table 4:** Efficiency analysis of IPN grading diagnosis by contrast-enhanced ultrasound.

**IPN Grade**	**Group**		**Total**
	**Experimental Control**		
I, II	13	35	48
III, IV	27	6	33
Total	40	41	81

**Table 5:** Analysis of diagnostic efficacy of vascular stenosis.

**Vascular Stenosis**	**Group**		**Total**
	**Experimental Control**		
No	13	26	39
Yes	27	15	42
Total	40	41	81

**Table 6:** Comparison of the diagnostic efficacy between contrast-enhanced ultrasound IPN classification and lumen stenosis.

**Methods**	**Diagnostic accuracy**	**Sensitivity**	**Specificity**	**Positive predictive value**	**Negative predictive value**
IPN	76.54	81.81	72.92	67.50	85.36
Vascular stenosis	65.43	64.29	66.67	67.50	63.41
x^2^	3.497	8.219	0.857	0	12.578
*P-value*	0.061	0.004	0.355	1	<0.001

**Table 7:** Logistic regression analysis of the influence of different factors on acute ischemic stroke.

**Variable**	**B**	**SE**	**Wald**	**OR**	**95% CI**	***P-*value**
IFN	1.149	0.181	40.141	3.156	2.212~4.503	<0.001
Vascular stenosis	-0.010	0.233	0.002	0.990	0.628~1.562	0.966
Age	0.240	0.124	3.734	1.271	0.997~1.622	0.053
Hypertension	1.031	0.459	5.058	2.81	1.14~6.90	0.025
Plaque length	0.013	0.018	0.503	1.013	0.977~1.622	0.478
Constant	-3.795	0.824	21.208	0.022		<0.001

## Data Availability

The data and supportive information are available within the article.

## LIST OF ABBREVIATIONS

AIS: Acute Ischemic Stroke
CEUS: Contrast-Enhanced Ultrasound
IPN: Intraplaque Neovascularization

## STANDARDS OF REPORTING

STROBE guidelines were followed.

## References

[1] Wang Y.J., Li Z.X., Gu H.Q., Zhai Y., Zhou Q., Jiang Y., Zhao X.Q., Wang Y.L., Yang X., Wang C.J., Meng X., Li H., Liu L.P., Jing J., Wu J., Xu A.D., Dong Q., Wang D., Wang W.Z., Ma X.D., Zhao J.Z. (2022). China stroke statistics: An update on the 2019 report from the National Center for Healthcare Quality Management in Neurological Diseases, China National Clinical Research Center for Neurological Diseases, the Chinese Stroke Association, National Center for Chronic and Non-communicable Disease Control and Prevention, Chinese Center for Disease Control and Prevention and Institute for Global Neuroscience and Stroke Collaborations.. Stroke Vasc. Neurol..

[2] Herpich F., Rincon F. (2020). Management of acute ischemic stroke.. Crit. Care Med..

[3] Li X., Li J., Wu G. (2021). Relationship of neutrophil‐to‐lymphocyte ratio with carotid plaque vulnerability and occurrence of vulnerable carotid plaque in patients with acute ischemic stroke.. BioMed Res. Int..

[4] Baud J.M., Stanciu D., Yeung J., Maurizot A., Chabay S., de Malherbe M., Chadenat M.L., Bachelet D., Pico F. (2021). Contrast enhanced ultrasound of carotid plaque in acute ischemic stroke (CUSCAS study).. Rev. Neurol..

[5] Musialek P., Rosenfield K., Siddiqui A.H., Grunwald I.Q. (2024). Carotid stenosis and stroke: Medicines, stents, surgery-"Wait-and-See" or Protect?. Thromb. Haemost..

[6] van Dam-Nolen D.H.K., Truijman M.T.B., van der Kolk A.G., Liem M.I., Schreuder F.H.B.M., Boersma E., Daemen M.J.A.P., Mess W.H., van Oostenbrugge R.J., van der Steen A.F.W., Bos D., Koudstaal P.J., Nederkoorn P.J., Hendrikse J., van der Lugt A., Kooi M.E. (2022). Carotid plaque characteristics predict recurrent ischemic stroke and TIA.. JACC Cardiovasc. Imaging.

[7] Han N., Ma Y., Li Y., Zheng Y., Wu C., Gan T., Li M., Ma L., Zhang J. (2023). Imaging and hemodynamic characteristics of vulnerable carotid plaques and artificial intelligence applications in plaque classification and segmentation.. Brain Sci..

[8] Zhou P., Shen Y., Wang L., Cao Z., Feng W., Liu J., Wang L., Meng P., Yang J., Xu W.Y., Gao P. (2020). Association between carotid intima media thickness and small dense low-density lipoprotein cholesterol in acute ischaemic stroke.. Lipids Health Dis..

[9] (2018). China's guidelines for the diagnosis and treatment of acute ischemic stroke 2018.. Chin. J. Neurol..

[10] Rafailidis V., Li X., Sidhu P.S., Partovi S., Staub D. (2020). Contrast imaging ultrasound for the detection and characterization of carotid vulnerable plaque.. Cardiovasc. Diagn. Ther..

[11] Song Y., Dang Y., Wang J., Cai H., Feng J., Zhang H., Ruan L. (2021). Carotid intraplaque neovascularization predicts ischemic stroke recurrence in patients with carotid atherosclerosis.. Gerontology.

[12] Wang Y.J., Li Z.X., Gu H.Q., Zhai Y., Jiang Y., Zhao X.Q., Wang Y.L., Yang X., Wang C.J., Meng X., Li H., Liu L.P., Jing J., Wu J., Xu A.D., Dong Q., Wang D., Zhao J.Z. (2020). China stroke statistics 2019: A report from the National Center for Healthcare Quality Management in Neurological Diseases, China National Clinical Research Center for Neurological Diseases, the Chinese Stroke Association, National Center for Chronic and Non-communicable Disease Control and Prevention, Chinese Center for Disease Control and Prevention and Institute for Global Neuroscience and Stroke Collaborations.. Stroke Vasc. Neurol..

[13] Chistiakov D.A., Orekhov A.N., Bobryshev Y.V. (2015). Contribution of neovascularization and intraplaque haemorrhage to atherosclerotic plaque progression and instability.. Acta Physiol..

[14] Schmidt C., Fischer T., Rückert R.I., Oberwahrenbrock T., Harms L., Kronenberg G., Kunte H. (2017). Identification of neovascularization by contrast-enhanced ultrasound to detect unstable carotid stenosis.. PLoS One.

[15] Xu J., Lu X., Shi G.P. (2015). Vasa vasorum in atherosclerosis and clinical significance.. Int. J. Mol. Sci..

[16] Lyu Q., Tian X., Ding Y., Yan Y., Huang Y., Zhou P., Hui P. (2021). Evaluation of carotid plaque rupture and neovascularization by contrast-enhanced ultrasound imaging: An exploratory study based on histopathology.. Transl. Stroke Res..

[17] Maitrias P., Metzinger-Le Meuth V., Nader J., Reix T., Caus T., Metzinger L. (2017). The involvement of miRNA in carotid-related stroke.. Arterioscler. Thromb. Vasc. Biol..

[18] Dzierwa K., Knapik M., Tekieli Ł., Mazurek A., Urbańczyk-Zawadzka M., Klecha A., Kowalczyk T., Koźmik T., Wiewiórka Ł., Banyś P., Węglarz E., Stefaniak J., Nizankowski R.T., Grunwald I.Q., Musiałek P. (2022). Clinical outcomes of extracranial carotid artery-related stroke eligible for mechanical reperfusion on top of per-guidelines thrombolytic therapy: Analysis from a 6-month consecutive patient sample in 2 centers.. Med. Sci. Monit..

[19] Shindo S., Fujii K., Shirakawa M., Uchida K., Enomoto Y., Iwama T., Kawasaki M., Ando Y., Yoshimura S. (2015). Morphologic features of carotid plaque rupture assessed by optical coherence tomography.. Am. J. Neuroradiol..

[20] Barnett H.J.M., Taylor D.W., Haynes R.B., Sackett D.L., Peerless S.J., Ferguson G.G., Fox A.J., Rankin R.N., Hachinski V.C., Wiebers D.O., Eliasziw M. (1991). Beneficial effect of carotid endarterectomy in symptomatic patients with high-grade carotid stenosis.. N. Engl. J. Med..

[21] Hermier M., Nighoghossian N., Adeleine P., Berthezène Y., Derex L., Yilmaz H., Dugor J.F., Dardel P., Cotton F., Philippeau F., Trouillas P., Froment J.C. (2003). Early magnetic resonance imaging prediction of arterial recanalization and late infarct volume in acute carotid artery stroke.. J. Cereb. Blood Flow Metab..

[22] Hammond C.J., McPherson S.J., Patel J.V., Gough M.J. (2008). Assessment of apparent internal carotid occlusion on ultrasound: Prospective comparison of contrast-enhanced ultrasound, magnetic resonance angiography and digital subtraction angiography.. Eur. J. Vasc. Endovasc. Surg..

[23] Saha S.A., Gourineni V., Feinstein S.B. (2016). The use of contrast-enhanced ultrasonography for imaging of carotid atherosclerotic plaques.. Neuroimaging Clin. N. Am..

[24] Brinjikji W., Huston J., Rabinstein A.A., Kim G.M., Lerman A., Lanzino G. (2016). Contemporary carotid imaging: From degree of stenosis to plaque vulnerability.. J. Neurosurg..

[25] Xiao J., Padrick M.M., Jiang T., Xia S., Wu F., Guo Y., Gonzalez N.R., Li S., Schlick K.H., Dumitrascu O.M., Maya M.M., Diniz M.A., Song S.S., Lyden P.D., Li D., Yang Q., Fan Z. (2021). Acute ischemic stroke versus transient ischemic attack: Differential plaque morphological features in symptomatic intracranial atherosclerotic lesions.. Atherosclerosis.

[26] Whelton P.K., Carey R.M., Aronow W.S., Casey D.E., Collins K.J., Dennison Himmelfarb C., DePalma S.M., Gidding S., Jamerson K.A., Jones D.W., MacLaughlin E.J., Muntner P., Ovbiagele B., Smith S.C., Spencer C.C., Stafford R.S., Taler S.J., Thomas R.J., Williams K.A., Williamson J.D., Wright J.T. (2018). 2017 ACC/AHA/AAPA/ABC/ACPM/AGS/APhA/ASH/ASPC/NMA/PCNA guideline for the prevention, detection, evaluation, and management of high blood pressure in adults: A report of the American College of Cardiology/American Heart Association Task Force on clinical practice guidelines.. Hypertension.

